# Proto-DS: A Self-Supervised Learning-Based Nondestructive Testing Approach for Food Adulteration with Imbalanced Hyperspectral Data

**DOI:** 10.3390/foods13223598

**Published:** 2024-11-11

**Authors:** Kunkun Pang, Yisen Liu, Songbin Zhou, Yixiao Liao, Zexuan Yin, Lulu Zhao, Hong Chen

**Affiliations:** Guangdong Key Laboratory of Modern Control Technology, Institute of Intelligent Manufacturing, Guangdong Academy of Sciences, Guangzhou 510070, China; kk.pang@giim.ac.cn (K.P.); sb.zhou@giim.ac.cn (S.Z.); yx.liao@giim.ac.cn (Y.L.); zx.yin@giim.ac.cn (Z.Y.); ll.zhao@giim.ac.cn (L.Z.); chen.h@giim.ac.cn (H.C.)

**Keywords:** hyperspectral imaging, adulteration, deep learning, self-supervised learning, data imbalance, prototypical network

## Abstract

Conventional food fraud detection using hyperspectral imaging (HSI) relies on the discriminative power of machine learning. However, these approaches often assume a balanced class distribution in an ideal laboratory environment, which is impractical in real-world scenarios with diverse label distributions. This results in suboptimal performance when less frequent classes are overshadowed by the majority class during training. Thus, the critical research challenge emerges of how to develop an effective classifier on a small-scale imbalanced dataset without significant bias from the dominant class. In this paper, we propose a novel nondestructive detection approach, which we call the Dice Loss Improved Self-Supervised Learning-Based Prototypical Network (Proto-DS), designed to address this imbalanced learning challenge. The proposed amalgamation mitigates the label bias on the most frequent class, further improving robustness. We validate our proposed method on three collected hyperspectral food image datasets with varying degrees of data imbalance: Citri Reticulatae Pericarpium (Chenpi), Chinese herbs, and coffee beans. Comparisons with state-of-the-art imbalanced learning techniques, including the Synthetic Minority Oversampling Technique (SMOTE) and class-importance reweighting, reveal our method’s superiority. Notably, our experiments demonstrate that Proto-DS consistently outperforms conventional approaches, achieving the best average balanced accuracy of 88.18% across various training sample sizes, whereas the Logistic Model Tree (LMT), Multi-Layer Perceptron (MLP), and Convolutional Neural Network (CNN) approaches attain only 59.42%, 60.38%, and 66.34%, respectively. Overall, self-supervised learning is key to improving imbalanced learning performance and outperforms related approaches, while both prototypical networks and the Dice loss can further enhance classification performance. Intriguingly, self-supervised learning can provide complementary information to existing imbalanced learning approaches. Combining these approaches may serve as a potential solution for building effective models with limited training data.

## 1. Introduction

Economically motivated adulteration is an inevitable occurrence in foods that are often intentionally substituted with inferior commercial varieties. The similarities in morphological characteristics, chemical constituents, and therapeutic activity between adulterated and genuine varieties contribute to the challenge of detection. Examples of such adulteration problems include citri reticulatae pericarpium (chenpi) [1], coffee beans [2], meats [3], oils [4], honey [5], seeds [6], and Chinese herbs [7]. Researchers have made significant efforts in proposing various detection methods to combat the issue of adulteration in recent years [8,9]. Among these methods, hyperspectral imaging has the advantages of rapid processing, nondestructive measurement, and less need for domain knowledge, making it suitable for online inspection [10,11,12].

The effectiveness of approaches based on Near-Infrared Reflectance (NIR) is heavily reliant on the performance of statistical analysis and machine learning techniques. For instance, Tankeu used partial least squares discriminant analysis (PLS-DA) to identify stephania tetrandra and aristolochia fangchi with hyperspectral imaging (HSI) [13]. Sun et al. demonstrated promising performance with PLS-DA for pinellia adulteration [14]. PLS-DA was also successfully applied to identify sorghum adulteration [15], and SVM was shown by Gomes et al. to be a better discriminative performance between special and traditional green coffee [16].

In addition to conventional machine learning algorithms, deep learning has also garnered significant interest from researchers due to its superior performance. Backhaus found that the radial basis function network yielded better performance and interpretability compared to MLP on coffee beans [17], while Feng showed reasonable performance with MLP in detecting adulterated honeysuckle tea leaves [18]. Liu et al. proposed a two-branch convolutional neural network (2B-CNN) to capture spatial–spectral information to address adulteration using datasets consisting of Chinese herbs, coffee beans, and strawberries [19]. Zheng et al. successfully distinguished the adulteration of minced mutton with a CNN [20], while Chakravartula et al. found that CNNs led to better performance in coffee adulteration prediction [21].

While the above methods show promise, they are primarily designed for balanced data distributions, which may not reflect real-world practical applications [22]. Acquiring a class-balanced dataset is a costly and time-consuming process, particularly when labeling unwanted substances is challenging. Additionally, conventional supervised learning algorithms often prioritize learning from the majority class, leading to neglect of the less frequent data during training. As a result, an effective system is required to handle datasets with small-scaled and skewed class proportions to ensure accurate true positive and true negative rates for the detector [23].

Current state-of-the-art approaches for addressing imbalanced learning in the context of spectroscopy can be categorized into two groups: oversampling the minority samples, and class-importance reweighting. One widely used oversampling approach is the Synthetic Minority Oversampling Technique (SMOTE), which generates synthetic data for the minority class through linear interpolation among the nearest samples. Amirruddin et al. [24,25] proposed LMTSmoteBoost, a combination of SMOTE and ensemble learning (i.e., Adaboost), demonstrating improved classification performance on imbalanced datasets. SMOTE is also compatible with deep learning models, such as Multi-Layer Perceptron (MLP) and CNN architectures, as demonstrated by Maktabi et al. [26] and Ozdemir [27]. Apart from oversampling, Wu et al. [28] proposed reweighting the class importance by considering the imbalance rates to train a CNN for rice seed vigor detection.

It is important to note that self-supervised learning is orthogonal to techniques for addressing imbalanced datasets, as both can be applied to either balanced or imbalanced learning problems. Self-Supervised Learning (SSL) aims to learn representations from unlabeled datasets by solving pretext tasks, thereby allowing subsequent tasks to benefit from the pretrained embeddings and improving generalization performance [29]. It has shown the ability to achieve performance comparable to supervised learning without requiring explicit labels [30,31,32,33]. Recent research has theoretically proven that SSL can reduce label bias from imbalanced datasets, allowing model performance to be further improved [34]. Later, Kotar showed that SSL can help models to learn reasonable representations from imbalanced data, sometimes slightly outperforming models trained on balanced data distributions [35]. In addition, Liu et al. demonstrated the potential of SSL to outperform supervised learning on imbalanced computer vision problems [36]. SSL can learn label-invariant and transferable features that improve the representation of rare classes and downstream tasks. However, there is limited research applying SSL to data imbalance problem involving hyperspectral imaging.

In this paper, we aim to address the imbalanced learning problem by combining the strengths of self-supervised learning (SSL), Dice loss, and prototypical networks. Our proposed model is trained in two steps. In Step 1, we introduce intra-instance spectral contrastive learning to the prototypical network, enabling it to learn sample-wise discriminative embeddings. In Step 2, the model is fine-tuned on the labeled imbalanced dataset using the Dice loss. Both steps ensure that the model is not biased towards the majority class and takes the minority class into account. This approach allows the trained model to effectively detect adulteration even when authentic products are mixed with a relatively small amount of counterfeit products. To demonstrate the effectiveness of our proposed method, we validate it on datasets consisting of images of chenpi, coffee beans, and Chinese herbs. The results show that our approach is more stable and robust compared to competitors across various imbalance ratios.

In summary, our contributions are as follows:

- We are the first to address the challenge of imbalanced data distribution in hyperspectral imaging-based nondestructive testing by incorporating self-supervised learning and Dice loss.
- We evaluate our approach on three imbalanced datasets, finding that it outperforms alternatives even in scenarios with extremely limited availability of minority samples.
- Our study reveals that self-supervised learning is key to realizing improved performance on imbalanced datasets. Additionally, combining self-supervised learning with the Dice loss further enhances model robustness.

## 2. Materials and Methods

### 2.1. Samples

**Figure 1:**
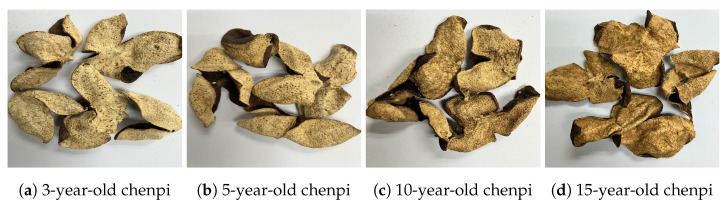

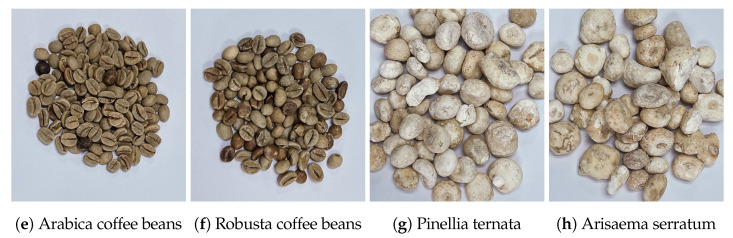
Figures of the imbalanced food products in the datasets: (**a**–**d**) samples from the chenpi dataset, (**e**,**f**) samples from the coffee bean dataset, and (**g**,**h**) samples from the Chinese herbs dataset.

To address the data imbalance problem, we curated three hyperspectral datasets: the chenpi dataset, the green coffee beans dataset, and the Chinese herbs dataset. These datasets contained samples that varied in price, quality, flavor, and effects, all of which were purchased from local supermarkets in Guangzhou, China. However, distinguishing these samples based on their colors, shapes, and contours is challenging, as shown in Figure 1. The chenpi dataset comprised dried peels of mature tangerines purchased from Xinhui County in Guangdong Province, China. chenpi must be dried and stored for at least three years to ensure high value and quality, with longer storage times believed to improve quality further. Due to sales and potential damage over time, the distribution of chenpi across different storage durations is naturally imbalanced. In this paper, we collected 1267 samples of 3-year-old chenpi, 197 samples of 5-year-old chenpi, 104 samples of 10-year-old chenpi, and 40 samples of 15-year-old chenpi. For the green coffee beans dataset, we collected 305 Arabica and 235 Robusta coffee beans. Arabica is often adulterated with Robusta due to the twofold price difference. Similarly, the Chinese herbs dataset contained 210 samples of Pinellia ternata and 203 samples of Arisaema serratum; Pinellia ternata is frequently adulterated with Arisaema serratum due to the difference in their cost. Unlike the green coffee bean and Chinese herb datasets, the chenpi dataset presents a unique challenge, requiring the model to distinguish between multiple classes. To simulate an imbalanced learning scenario, we experimented with various imbalance rates, which are detailed in Section 2.5.

### 2.2. Hyperspectral System and Acquisition of Spectra

Hyperspectral data for chenpi, coffee beans, and Chinese herbs were acquired using two NIR hyperspectral imaging devices (SPECIM, Spectral Imaging Ltd., Oulu, Finland) with a diffraction grating and an InGaAs sensor matrix. To create the coffee bean and Chinese herb datasets, we used a line-scan hyperspectral camera (N17E) with a spectral range of 900–1700 nm, capturing 256 bands; the frame rate and exposure time were set to 21 Hz and 28.3 ms, respectively. For the Chenpi dataset, we used a Specim FX17 hyperspectral camera, also covering the 900–1700 nm spectral range but with 224 bands, a frame rate of 234 Hz, and an exposure time of 4.2 ms.

Upon scanning the object with the hyperspectral camera, three-dimensional data cubes are automatically generated. Each hyperspectral data cube has a spatial resolution of 320 × 640 pixels, with random sample positioning applied across all datasets. To extract the Region of Interest (ROI), we used the watershed algorithm to detect the object’s contour. Based on the center of the ROI, we cropped the object from the raw hyperspectral image into square regions: 192 × 192 pixels for chenpi, 40 × 40 pixels for coffee beans, and 60 × 60 pixels for Chinese herbs. Subsequently, the segmented hyperspectral image, denoted as $I_{o}$, underwent min–max normalization to rescale the image as $I_{c}$:

(1) $$\begin{array}{c} \begin{array}{c} I_{c}=\frac{I_{o}-I_{D}}{I_{W}-I_{D}} \end{array} \end{array}$$

where $I_{D}$ and $I_{W}$ are the dark and white reference images, respectively. Because hyperspectral images may contain pixels with the poor-quality signals (e.g., dead pixels, dark pixels with spike spectra, or objects under occlusion shadows), the pixels were considered to be effective if the average value of particular pixel was larger than 0.1. Unlike other CNN approaches that learn to extract the texture and spatial information for detection, we use the spectral data directly, as the objects’ differences in shape and texture are less meaningful for food adulteration. During training, we insisted on using all effective NIR spectra rather than the averaged spectra for every product, resulting in a larger training set. However, as the original hyperspectral data could exhibit a relatively low signal-to-noise ratio at both the beginning and end of the spectra [33,37,38], we retained the spectra ranging from 1000 to 1600 nm to make for a more robust representation. In the following section, we denote the preprocessed NIR spectra as $x\in R^{D}$ and the class labels as $y_{i}\in \left\{1,\cdots ,C\right\}$

### 2.3. Proposed Method

**Figure 2:**
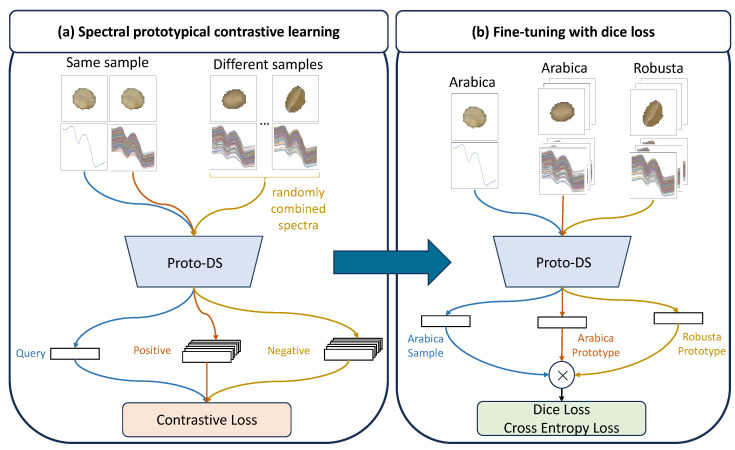
Training process of Proto-DS using spectral prototypical contrastive learning and fine-tuning with Dice loss to improve the prototypical network. For simplicity, we use the coffee bean dataset as an example. Blue line: data flow of unknown new incoming samples. Red line:the data flow of the positive samples (majority class). Yellow line: the data flow of the negative samples (minority class).

Overall, our Proto-DS method, combines the strengths of Dice loss, self-supervised learning, and prototypical networks to address the challenges of learning from small-scaled imbalanced datasets. Given that the pretextual task of self-supervised learning may not be directly relevant to the successive classification task, our model undergoes a two-step training process, as depicted in Figure 2. Spectral prototypical contrastive learning: First, the model is pretrained using the proposed instance-wise spectra prototypical contrastive learning to acquire a richer feature representation without using supervision. Fine-tuning with Dice loss: Second, we construct a classifier to distinguish majority and minority samples *f* by fine-tuning the learned representation on the labeled datasets using the Dice loss.

Here, we first discuss the adopted network architecture in Section 2.3.1, then discuss the self-supervised learning and fine-tuning with Dice loss schemes in Section 2.3.2 and Section 2.3.3, respectively.

#### 2.3.1. Prototypical Network Architecture

The metric learning-based approach demonstrates strong potential for generating effective representations when training samples are limited, offering robustness even with smaller datasets. By reducing the inter-class similarity and increasing the intra-class dissimilarity, metric learning proves more effective than traditional classification models [39], which often overfit, especially when the majority class dominates the training process. While metric-learning methods may not always outperform traditional models on balanced datasets, this is not the focus of our paper.

In this study, we explore the original prototypical network in the context of hyperspectral datasets with skewed class distributions, a gap that forms the central focus of our work. We train a metric learning-based prototypical network using spectral prototypical contrastive learning, followed by fine-tuning with the Dice loss. The prototypical network is designed to learn embeddings that transform the data, enabling recognition through a fixed nearest-neighbor classifier [40,41].

The prototypical network with a set pooling layer extracts a vectorized representation $c_{k}\in R^{M}$ (prototype) for each class by averaging their training samples on the learned embedding space $f_{\phi }:R^{D}\to R^{M}$:

(2) $$\begin{array}{c} \begin{array}{c} c_{k}=\frac{1}{|y_{k}|}\sum_{\left(x_{i},y_{i}\right)\in y_{k}}f_{\phi }\left(x_{i}\right). \end{array} \end{array}$$

**Figure 3:**
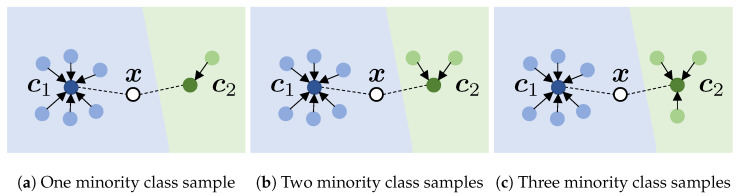
The prototypical network with various available samples for training. The blue color indicates that the object is labeled as an authentic sample, while the green color indicates that the object is labeled as counterfeit. The light blue and light green data points denote the training samples in the embeddings. whereas the dark blue and dark green data points indicate the prototypes for authentic $c_{1}$ and counterfeit $c_{2}$, respectively. The white circle indicates the unknown test data, while the dashed line represent the distance to the prototype vectors.

As Figure 3 demonstrates, the sample size invariance of the set pooling layer is designed to convert the given samples into a prototype regardless of the sample size.

Next, the unknown test data are classified as authentic or counterfeit based on the softmax Euclidean distance to the closest prototype vectors $c_{k}$. The reason for using the Euclidean distance is that it is generally considered more robust than a learned nonlinear metric or the cosine distance [40].

(3) $$\begin{array}{c} \begin{array}{c} p_{\phi }\left(y=k|x\right)=\frac{exp(-d\left(f_{\phi }\left(x\right),c_{k}\right)))}{\sum_{k^{\prime }}exp\left(-d\left(f_{\phi }\left(x\right),c_{k}^{\prime }\right)\right))} \end{array} \end{array}$$

To fine-tune this network, a subset of training NIR spectra from each class were randomly subsampled and used to extract the prototype vector $c_{k}$ for every epoch. Then, all training data served as the query sample to compute the softmax probability via Equation (3). Unlike the original prototypical network, which directly minimizes the negative log probability of the true class *k*, we instead optimized an improved version for imbalanced learning. The details are provided in the Section 2.3.3.

Our approach incorporates four stacked blocks, each consisting of a batch normalization layer, a fully connected layer, and a Leaky ReLU activation function. Unlike the original prototypical network, we follow the architecture proposed by Liu et al. and reposition the batch normalization layer to rescale the input before passing it to the fully connected layer [19,42]. Through empirical analysis, we have observed that incorporating the batch normalization layer helps to stabilize the training and improves generalization with NIR data. The details of the architecture are provided in Table 1.

#### 2.3.2. Spectral Prototypical Contrastive Learning

We propose an instance-wise spectral prototypical contrastive learning approach combining a self-supervised pretext task and a prototypical network specifically tailored to the limited dataset size of NIR spectra data. The underlying concept of instance-wise contrastive learning is to learn an embedding where similar pairs are grouped closely together while dissimilar pairs are pushed apart. In our hypothesis, spectra within the same instance should exhibit similarity while differing significantly from spectra of other examples.

To achieve this, we define positive pairs as effective pixels’ spectra originating from the same instance, while negative pairs are randomly created from both the spectra of the particular instance and spectra of other samples. This formulation enables the model to learn a richer feature representation for distinguishing individual samples, which benefits the subsequent imbalanced classification task. By starting with a meaningful representation, we reduce the search space during supervised fine-tuning and mitigate the risk of heavy bias towards the majority class. Therefore, the loss function of our contrastive learning approach can be expressed as follows:

(4) $$\begin{array}{c} \begin{array}{c} \ell_{SSL}=-\log\frac{\exp(d\left(f_{\theta }\left(x_{i}^{q}\right),f_{\theta }\left(x_{i}^{+}\right)\right)/\tau }{\exp(d\left(f_{\theta }\left(x_{i}^{q}\right),f_{\theta }\left(x_{i}^{+}\right)\right)/\tau +\exp(d\left(f_{\theta }\left(x_{i}^{q}\right),f_{\theta }\left(x_{i}^{-}\right)\right)/\tau } \end{array} \end{array}$$

where $d:R^{M}\times R^{M}\to \left[0,+\infty \right)$ represents a distance function between two NIR spectra, *B* is the size of the negative pair set, and τ denotes the temperature parameter of the softmax distribution. Here, we set the size of negative pair B=16 and temperature τ=1 for the experiment.

#### 2.3.3. Fine-Tuning with Dice Loss

We construct the classifier *f* for the imbalanced classification task based on the self-supervised learned representation mentioned earlier. However, this requires two main issues to be addressed: (1) the pretext task of self-supervised learning may not directly relate to the successive classification task, and (2) the model might be heavily biased by the majority class. To overcome the first issue, we fine-tuned the pretrained model on the imbalanced dataset using a supervised learning approach with labeled samples. This fine-tuning process involved updating the pretrained model parameters with additional epochs, leveraging both self-supervised pretrained knowledge and discriminative knowledge.

To address the second issue, we first note that imbalanced learning problems often exhibit skewed class proportions, which can cause the majority class to dominate the model training process, resulting in neglect of the minority data. One effective approach is to train the model with an objective function that balances the bias between the majority and minority classes, allowing the model to avoid overconfidence in the majority class. The Dice loss, initially designed to address the challenge of imbalanced distributions between foreground and background pixels in semantic segmentation [43], can be particularly useful here:

(5) $$\begin{array}{c} \begin{array}{c} \ell_{Dice}=\frac{1}{C}\sum_{c}^{C}\left(1-\frac{2\sum_{i}p_{i}y_{i}}{\sum_{i}p_{i}^{2}+\sum_{i}y_{i}^{2}}\right) \end{array} \end{array}$$

in the equation, $\frac{2\sum_{i}p_{i}y_{i}}{\sum_{i}p_{i}^{2}+\sum_{i}y_{i}^{2}}$ corresponds to the Dice–Sørensen coefficient (DSC), while $p_{i}$ and $y_{i}$ represent the predicted probability and one-hot ground truth label, respectively; more specfically, the DSC is an F1-oriented statistic that measures the similarity between two sample sets.

(6) $$\begin{array}{c} \begin{array}{c} DSC=\frac{2TP}{2TP+FN+FP}=\frac{2\frac{TP}{TP+FN}\frac{TP}{TP+FP}}{\frac{TP}{TP+FN}+\frac{TP}{TP+FP}}=\frac{2\times Precision\times Recall}{Precision+Recall}=F1 \end{array} \end{array}$$

Optimizing the loss function based on the DSC disregards the true negative, preventing the majority class from dominating the training [44,45]. Unlike methods based on resampling or reweighting methods, which require prior adjustments to the training data distribution based on the imbalance ratio, the Dice loss can handle imbalanced learning without the need for this ratio. In this paper, we enhance the original objective function of the prototypical network by incorporating both the Dice and cross-entropy losses.

### 2.4. Implementation Details

**Figure 4:**
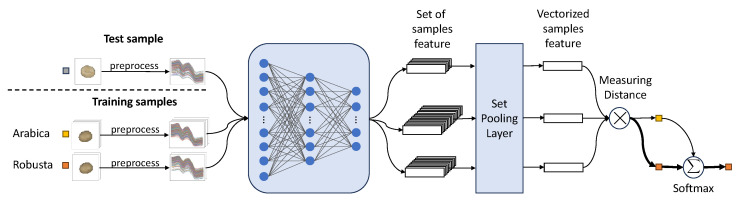
The test process of Proto-DS. Light grey box: unknown new incoming samples during testing. Yellow box: training for Arabica coffee beans (majority class). Orange box: training for Robusta coffee beans (minority class).

To train Proto-DS, we utilized the Adam optimizer with a learning rate of $1\times 10^{-4}$ for both self-supervised learning and fine-tuning, where the self-supervised learning step was optimized with 200 epochs and the fine-tuning step with 50 epochs, respectively. Subsequently, prototypes were extracted from the training set using the final fully trained model. Specifically, we treated each effective pixel’s spectrum from the same instance as a noisy spectrum, resulting in a larger training sample size than using the averaged spectra alone during training. As shown in Figure 4, during testing, we employed a set pooling layer to average the hidden representations of all spectra within an instance, producing a vector representation that enables the hyperspectral data to benefit from the power of Proto-DS. Then, we classified the unknown test sample by comparing the similarity between the vector representation and the training prototypes.

### 2.5. Experiment Settings

We evaluated the performance of all algorithms with various imbalance rates. First, we split the datasets into training and test sets to evaluate the performance of all algorithms under imbalanced conditions. For the chenpi dataset, we split the data evenly, with 50% allocated for training and 50% for testing. For the coffee bean and Chinese herb datasets, we used 80% of the data for training and the remaining 20% for testing.

**Figure 5:**
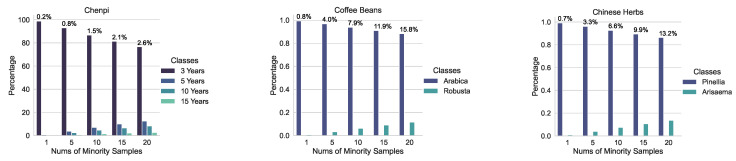
Class distributions for the coffee bean and Chinese herb datasets; dark blue denotes the majority class, light blue denotes the minority class, and percentage indicates the imbalance rate for the specific imbalance setting. Please note that the Chenpi dataset contains multiple minority classes, which we represent using different light blue colors.

Next, to create datasets with varying imbalance rates, we adjusted the number of available minority class samples in each subset. Specifically, while keeping the majority class sample size constant, we varied the size of the minority class samples in the training subsets as follows: n=1,5,10,15,20. For the chenpi dataset, which naturally contained multiple minority classes (i.e., 5-year-old, 10-year-old, and 15-year-old chenpi), we set the sample size of the least represented minority class (15-year-old chenpi) to n=1,5,10,15,20. The sample sizes for the 5-year-old and 10-year-old chenpi were adjusted according to their original ratios relative to the 15-year-old chenpi. The size of the 3-year-old chenpi (the majority class) was kept constant across all imbalance rates. This approach resulted in various imbalance rates for the chenpi, coffee bean, and Chinese herb datasets, as illustrated in Figure 5.

### 2.6. Evaluation Metrics

Generally, the evaluation metric of accuracy is commonly used in balanced classification tasks. However, in the context of imbalanced scenarios accuracy can be misleading, as it is heavily influenced by the majority class; moreover, a single universally applicable evaluation metric that suits all types of imbalanced label distributions is lacking. Thus, in this paper we apply multiple performance metrics, namely, balanced accuracy (B.Acc) [23], macro-average F1-score (M.F1-Score) [46], macro-average area under the curve of the receiver operating characteristic (M.AUROC) [47], and macro-average precision (M.AP) [48], to assess the overall performance of the models. Additionally, we utilize the sensitivity (Sens.) and specificity (Spec.) to evaluate the behavior of the models. It is important to note that the macro metrics (M.F1-Score, M.AUROC, M.AP) are averaged across classes. This is done to ensure that equal importance is assigned to both classes in the imbalanced detection problem. Similarly, the motivation behind calculating the balanced accuracy is to average the intra-class accuracy, which is equivalent to averaging both sensitivity and specificity in binary classification.

### 2.7. Methods for Comparison

**LMT-S [24]:** For comparison, we used the conventional machine learning approach LMTSmoteBoost (LMT-S), which combines logistic model trees with SMOTE and Adaboost. LMT-S has demonstrated promising classification performance on imbalanced datasets. In our experiment, we adopted the default settings of LMT, SMOTE, and Adaboost, which align with the default Weka configuration.

**MLP-S [26]:** In addition to the conventional machine learning approach, we compared our method with a related deep learning approach that incorporates the SMOTE technique. In this approach, a Multi-Layer Perceptron (MLP) is used instead of logistic model trees and Adaboost. Specifically, MLP-S employs two fully connected layers and utilizes the leaky ReLU activation function. To train the MLP-S model, we used the cross-entropy loss with uniform class weight and the default settings of SMOTE.

**Conv-S [27]:** This approach replaces the fully connected layers of MLP-S with a 1D convolutional layer. Additionally, Conv-S includes max-pooling layers after each leaky ReLU function. Similarly to MLP-S, Conv-S optimizes the vanilla cross-entropy loss and increases the size of the minority group by following the SMOTE settings proposed by Ozdemir et al. [27].

**Conv-W [28]:** In contrast to oversampling approaches, deep learning techniques can also adjust the class importance during optimization by reweighting, thereby reducing the influence of heavily-tailed distributions. To achieve this, we followed the method of Wu et al. for incorporating class weights into the model [28]. Furthermore, we disabled SMOTE during training to ensure that the CNN could benefit only from the scheme for reweighting the class importance.

**MLP-W:** To maintain consistency, we applied the same scheme for reweighting the class importance to the Multi-Layer Perceptron (MLP) model (MLP-S). To examine the effectiveness of the reweighting scheme, we retained the same architecture as in MLP-S but disabled SMOTE.

## 3. Results

### 3.1. Analysis of Spectra

**Figure 6:**
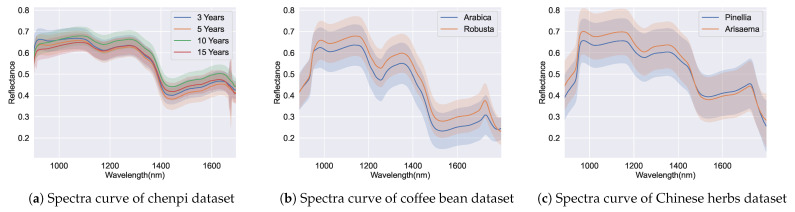
Visualisation spectra of each dataset. The straight lines are the averaged spectra among the particular classes, while the shaded area indicates the standard deviation of each class.

The range of the spectra, which is crucial for hyperspectral imaging applications, requires researchers to identify the effective range of the spectral band before applying machine learning techniques. We visualized the averaged spectra with the corresponding standard deviation for all datasets. In Figure 6, the shaded areas at the beginning and end of the wavelength range exhibit significant overlaps, while the middle range is more distinguishable, suggesting that the chosen 1000–1600 nm range provides more separable information for the model to learn from compared to the full 900–1700 nm range. Additionally, the differences in the shaded area between the samples of the majority class and minority class are barely identifiable when directly using the features of the spectra, as they have similar spectral signatures and variability. This implies that a nonlinear classification model might be better suited for this task.

### 3.2. Comparison with Baselines

**Figure 7:**
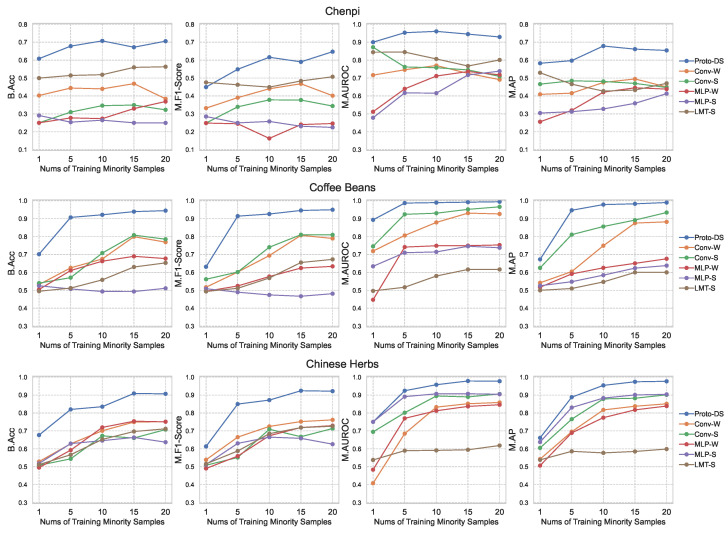
Comparison with other state-of-the-art competitors in terms of balanced accuracy (B.Acc), M.F score (Macro-F score), macro-AUROC (M.AUROC), and macro-average precision (M.AP). **Proto-DS**: proposed method; **Conv-W**: CNN with class-reweighted cross entropy loss; **Conv-S**: CNN with SMOTE; **MLP-W**: MLP with class-reweighted cross entropy loss; **MLP-S**: MLP with SMOTE; **LMT-S**: logistic model tree with SMOTE and Adaboost.

**Figure 8:**
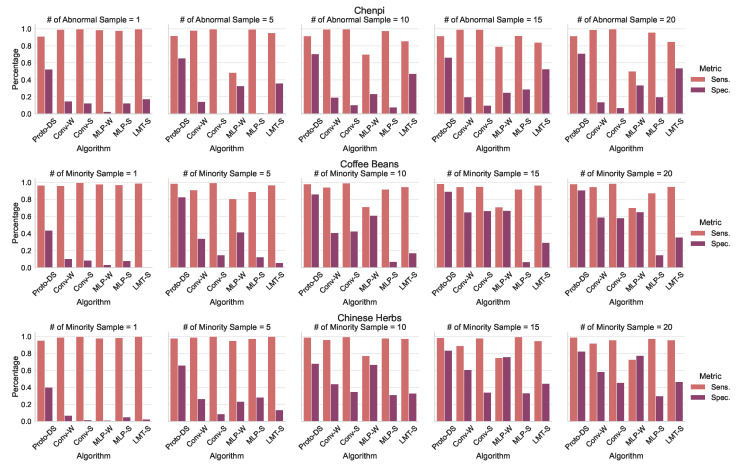
Comparison with state-of-the-art competitors in terms of sensitivity and specificity. Each figure summarizes the class-wise performance for all algorithms, while the rows corresponding to the different datasets.

To assess the effectiveness of Proto-DS, we conducted a comprehensive analysis by comparing it with other baselines in terms of two aspects: overall performance (as shown in Figure 7) and class-wise performance (as illustrated in Figure 8). The overall performance captures quantified statistics of the models’ performance across both classes, including metrics such as B.Acc, M.Fscore, M.AUROC, and M.AP. On the other hand, the class-wise performance provides insights into the models’ behavior by examining their performance on individual classes.

The first takeaway from the overall performance results in Figure 7 is that our proposed Proto-DS consistently outperforms other state-of-the-art methods across all datasets irrespective of the imbalance rate. Proto-DS demonstrates strong performance on the chenpi dataset, achieving a balanced accuracy (B.Acc) of 60.81% with just one minority training sample and an impressive 70.57% with 20 minority samples. Similarly, Proto-DS achieves a balanced accuracy ranging from 70.14% to 94.46% and 67.72% to 90.69% with a range of 1–20 minority samples on the coffee bean and Chinese herb datasets, respectively. While there are subtle performance variations among the imbalanced methods employed for the MLP and CNN models, implementation of the Proto-DS approach yields a remarkable performance benefit in terms of balanced accuracy (B.Acc), even in scenarios characterized by highly severe class imbalances (i.e., one minority sample). Importantly, even when the Proto-DS model is trained with only five minority samples (representing imbalance rates of 0.8%, 3.3%, and 4.0%, respectively), it still demonstrates superior performance compared to both the CNN-based and MLP-based imbalanced learning approaches trained with even more minority data (e.g., 10/15/20). These observations empirically demonstrate the superior appeal of our proposed approach compared to conventional imbalanced deep learning methods for handling spectroscopy data.

Notably, it is worth highlighting that the LMTSmoteBoost method performs the poorest among all deep learning methods on the coffee bean and Chinese herb datasets, but has the second-best performance on the chenpi dataset. This finding suggests that deep learning approaches can potentially learn a better representation without the need for labor-intensive feature engineering. However, these models can easily become biased by the majority class and confuse the decision boundary, leading to poorer and less robust performance on multiclass and imbalanced data, such as the chenpi dataset in our case.

Figure 8 presents the class-wise performance of all algorithms on both datasets with varying imbalance rates. While the sensitivity of Proto-DS does not always surpass that of all competitors, it consistently exhibits the highest specificity compared to other methods. Of particular importance, Proto-DS demonstrates reasonable specificity even in severely imbalanced scenarios such as those with only one minority training sample. In contrast, the competing methods experience significantly worse performance in such cases. These findings suggest that traditional approaches such as SMOTE and class reweighting schemes are not advantageous for extremely imbalanced scenarios, as they suffer from severe overfitting issues, specifically overfitting to the majority class. Conversely, our pretrained Proto-DS leverages self-supervised learning, which mitigates the overfitting problem by learning meaningful representations. This advantage allows Proto-DS to maintain robust performance even in cases of extreme imbalance.

## 4. Discussion

### 4.1. Contributions of the Proposed Components

We conducted an ablation study to assess the contributions of the different components in our proposed method. Specifically, we evaluated the Proto-DS model without Dice loss (w/o D) and without self-supervised learning (w/o SSL) as well as the vanilla prototypical network (w/o SSL + D). Each model was retrained from scratch with the corresponding component removed.

**Figure 9:**
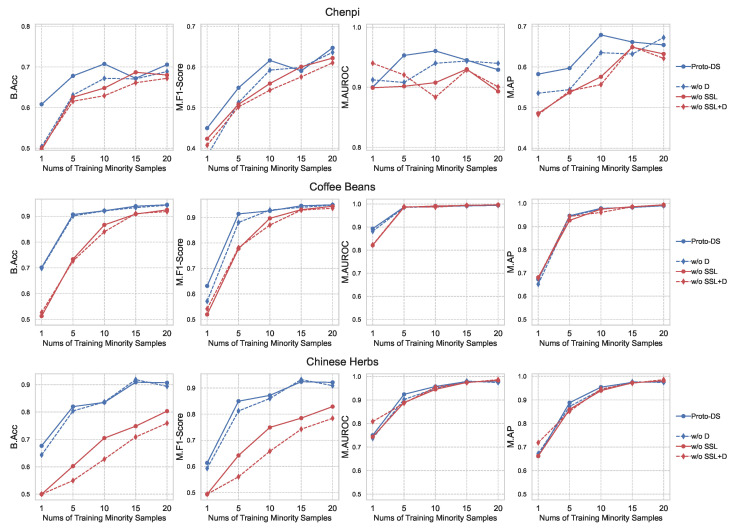
Results of the ablation study comparing different components in terms of balanced accuracy (B.Acc), M.F score (M.F1-score), macro-AUROC (M.AUROC), and macro-average precision (M.AP): **Proto-DS**, proposed method (blue straight line); **w/o D**, without applying Dice loss (blue dashed line); **w/o SSL**, without applying self-supervised learning (red straight line); **w/o SSL + D**, without self-supervised pretraining or Dice loss (red dashed line).

**Figure 10:**
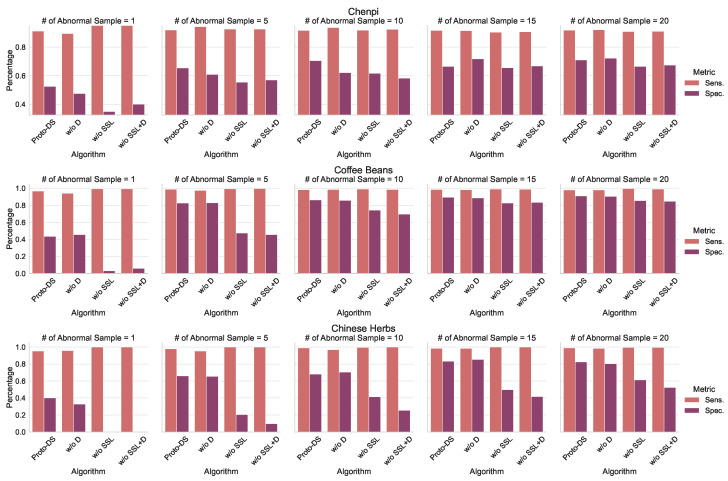
Results of the ablation study comparing different components in terms of sensitivity (Sens.) and specificity (Spec.): **Proto-DS**, proposed method; **w/o D**, without applying Dice loss **w/o SSL**, without applying self-supervised learning; **w/o SSL + D**, without self-supervised pretraining or Dice loss.

As shown in Figure 9 and Figure 10, the models pretrained with self-supervised learning demonstrate a significant performance boost across both datasets, particularly in terms of B.Acc and M.F1. This substantial improvement highlights self-supervised learning as the most impactful component in our method. By leveraging spectral prototypical contrastive learning, the model gains richer representations that integrate knowledge from prototypical supervised learning. This allows the pretrained model to be fine-tuned more effectively from a similar representation, reducing the risk of overfitting on the majority class. Additionally, we observed that incorporating the Dice loss further enhances model performance. Incorporating the Dice loss directly optimizes performance in imbalanced learning, encouraging the model to focus more on the minority class. This leads to further improvements, especially when combined with pretrained self-supervised learning.

### 4.2. Intuition of the Contributed Components

**Figure 11:**
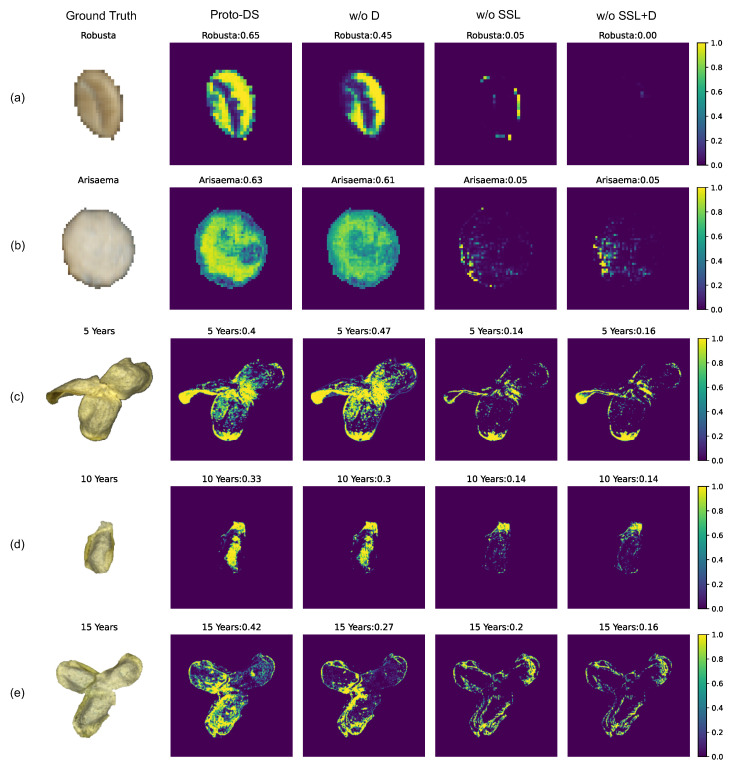
Visualization of the proposed model’s pixel-level probability for the corresponding class on various datasets. (**a**) Robusta (**b**) Arisaema (**c**) 5-Year-old Chenpi (**d**) 10-Year-old Chenpi (**e**) 15-Year-Old Chenpi. The rows represent particular samples from the minority class, while the columnsrepresent the raw image (Ground Truth), proposed method with all components (Proto-DS), and Proto-DS without particular components (w/o D, w/o SSL., w/o SSL + D). Brighter pixels indicate high probability on the corresponding class, while **darker pixels** indicate low probability on the corresponding class.

In this section, we delve into the intuition behind these contributed components by visualizing the predictions of the corresponding models and exploring the complementary nature of self-supervised learning and Dice loss. Figure 11 demonstrates our analysis of how these different components affect the prototypical network’s accurate prediction of pixel samples.

Upon analyzing the probability distribution of the minority samples in Figure 11 (indicated by brighter areas), a clear distinction emerges between the models with and without spectral prototypical contrastive learning (w/o SSL, w/o SSL + D). The models without this learning technique have lower confidence on the corresponding class; conversely, the model incorporating spectral prototypical contrastive learning (Proto-DS, w/o D) exhibits a significantly higher level of agreement in identifying minority class samples. Notably, the model with self-supervised learning displays the ability to consider a broader area of the object when identifying the minority sample. This suggests that self-supervised learning enables the model to extract information at the instance level rather than solely relying on pixel-level analysis. Such an approach highlights the importance of spectral prototypical contrastive learning, as it empowers the model to acquire representations that are less sensitive to variations in individual pixel reflectance intensities.

Our analysis of the Dice loss in Figure 11b–e reveals several interesting insights. According to the figure, the Dice loss appears to contribute equally with the original cross-entropy loss when the model is trained without self-supervised pretraining. However, when applied to a model pretrained with self-supervision, the Dice loss produces a smoother and broader confidence area around the target object. This suggests that the Dice loss helps the model to become more confident in identifying shadow regions characterized by low reflectance, such as edges, shapes, and depressions. One possible explanation for this is that spectra in low-reflectance areas are more sensitive to variations caused by irradiation angles, resulting in higher variance and making these regions more challenging for the model to learn. Interestingly, the Dice loss also promotes smoother agreement across effective pixels, not only enhancing confidence in low-reflectance areas but also mitigating overconfidence in high-reflectance regions. As a result, the Dice loss improves the robustness of hyperspectral imaging models in handling imbalanced learning problems by reducing overconfidence in heavily biased pixel spectra.

### 4.3. Two-Dimensional Visualization of the Proto-DS Learned Space

This investigation focuses on understanding how the proposed method enhances the performance of imbalanced learning by visualizing the representations in 2D space. To achieve this, we employed PCA to reduce the dimensionality of the final hidden layer, followed by fitting a 2D Gaussian distribution for each class. Subsequently, we evaluated the separability of these distributions by measuring the Intersection Over Union (IOU).

**Figure 12:**
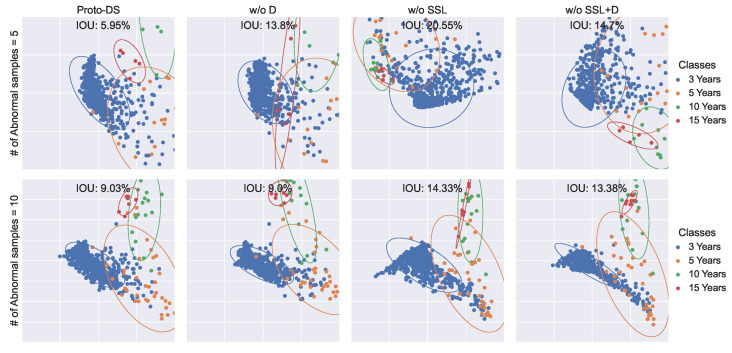

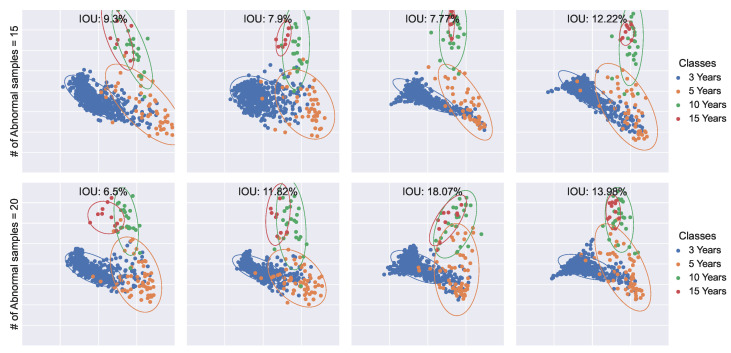
Two-dimensional visualization of the learned feature space for the chenpi dataset with multiple settings: the rowsrepresent various minority training sample sizes (5/10/15/20), while the columns represent the proposed method with all components (Proto-DS) or without particular components (w/o S., w/o F. + S., w/o SSL + S., w/o SSL + F. + S.).

**Figure 13:**
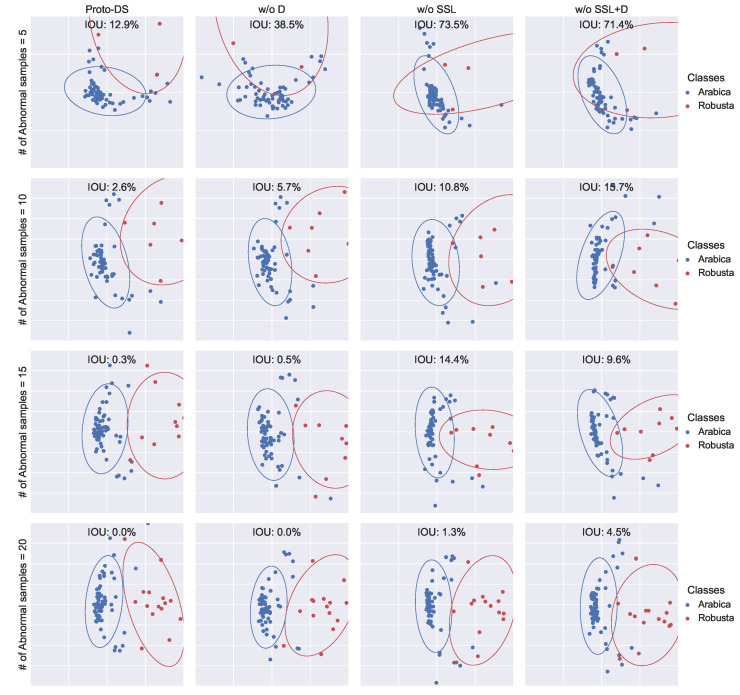
Two-dimensional visualization of the learned feature space for the coffee bean dataset with multiple settings: the rows represent various minority training sample sizes (5/10/15/20), while the columns represents the proposed method with all components (Proto-DS) or without particular components (w/o S., w/o F. + S., w/o SSL + S., w/o SSL + F. + S.).

**Figure 14:**
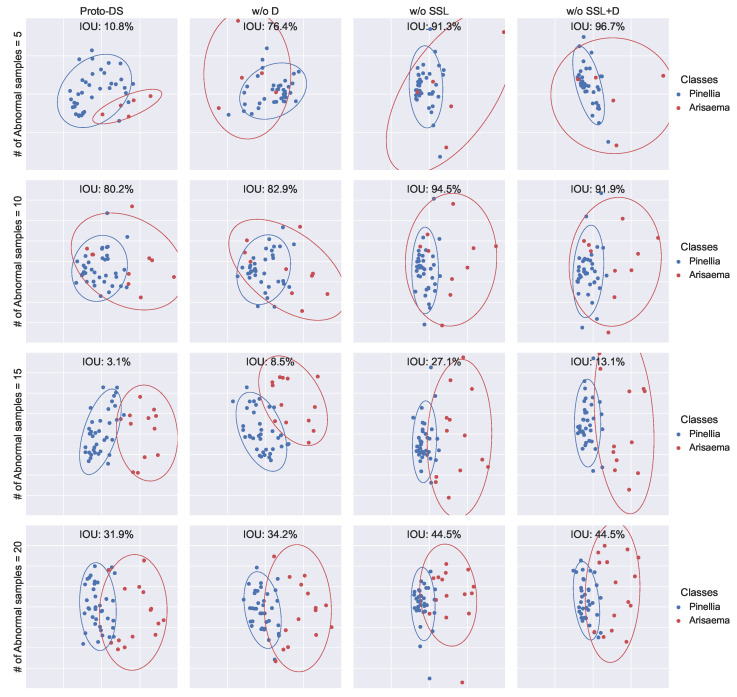
Two-dimensional visualization of the learned feature space for the Chinese herbs dataset with multiple settings: the rows represent various minority training sample sizes (5/10/15/20), while the columns represent the proposed method with all components (Proto-DS) or without particular components (w/o S., w/o F. + S., w/o SSL + S., w/o SSL + F. + S.).

Based on the findings presented in Figure 12, Figure 13 and Figure 14, Proto-DS consistently exhibits the most separable representation with the minimum IOU. As components are progressively removed, the hidden representation becomes less separable. Notably, removing all components results in the poorest separability, as seen in the vanilla prototypical network without SSL and Dice loss (w/o SSL + D).

When the Dice loss is employed (w/o SSL), the IOU area decreases significantly, indicating improved separability. Intriguingly, the combination of Dice loss and self-supervised learning further enhances the separability of the hidden space (w/o S.). This phenomenon becomes even more pronounced when combined with SMOTE in Proto-DS. These observations substantiate the finding that each of the proposed components provides complementary information that collectively improves performance on imbalanced data.

### 4.4. Limitations and Future Work

Our work has several limitations and opportunities for future improvement. First, the proposed method is primarily designed for classification tasks. A natural extension would be to adapt the framework for regression tasks (e.g., detecting milk adulteration), which would broaden its applicability to various food adulteration problems. Additionally, only one age-sensitive food dataset was evaluated. It would be valuable to assess the method’s effectiveness on other challenging age-sensitive datasets. In the future, we would like to explore the proposed framework to support soft labels (i.e., regression tasks) and inspire further innovations in nondestructive testing approaches with fewer constraints on dataset collection.

## 5. Conclusions

In this paper, we have proposed a novel approach for addressing data imbalance in hyperspectral image data by combining self-supervised learning, Dice loss, and prototypical networks. Our key success consists of self-supervised learned representations with spectral prototypical contrastive learning, resulting in robust generalization performance that surpasses conventional methods. Furthermore, empirical analyses on the coffee bean and Chinese herb datasets validate the effectiveness of spectral prototypical contrastive learning and Dice loss, which complement each other to significantly improve model performance. We envision that this combination can be a cornerstone for building effective models despite inadequate training data. Moreover, it enhances the potential of nondestructive testing using hyperspectral imaging for broad application to age-sensitive food products.

## Figures and Tables

**Table 1:** Architecture of the prototypical network.

Layer	Input Dimension	Output Dimension	Activation Function
Batchnorm Layer 1	192	192	N/A
Linear Layer 1	256	256	LeakyReLU
Batchnorm Layer 2	256	256	N/A
Linear Layer 2	256	256	LeakyReLU
Batchnorm Layer 3	256	256	N/A
Linear Layer 3	256	256	LeakyReLU
Batchnorm Layer 4	256	256	N/A
Linear Layer 4	256	256	LeakyReLU
Set Pooling Layer	N/A	N/A	N/A

## Data Availability

The original contributions presented in the study are included in the article, further inquiries can be directed to the corresponding author.
